# Real-Life Retention Rates and Reasons for Switching of Biological DMARDs in Rheumatoid Arthritis, Psoriatic Arthritis, and Ankylosing Spondylitis

**DOI:** 10.3389/fmed.2021.708168

**Published:** 2021-09-27

**Authors:** Vandana Bhushan, Susan Lester, Liz Briggs, Raif Hijjawi, E. Michael Shanahan, Eliza Pontifex, Jem Ninan, Catherine Hill, Fin Cai, Jennifer Walker, Fiona Goldblatt, Mihir D. Wechalekar

**Affiliations:** ^1^Rheumatology Unit, Flinders Medical Centre, Adelaide, SA, Australia; ^2^Division of Medicine, Flinders Medical Centre, Adelaide, SA, Australia; ^3^Rheumatology Unit, Queen Elizabeth Hospital, Adelaide, SA, Australia; ^4^Discipline of Medicine, The University of Adelaide, Adelaide, SA, Australia; ^5^College of Medicine and Public Health, Flinders University, Adelaide, SA, Australia

**Keywords:** biologic DMARDs, retention, switching, rheumatoid arthritis, psoriatic arthritis, ankylosing spondylitis

## Abstract

**Aims:** To determine real-life biologic/targeted synthetic disease-modifying anti-rheumatic drug (b/tsDMARD) retention rates in rheumatoid arthritis (RA), psoriatic arthritis (PsA), and ankylosing spondylitis (AS), explore reasons for switching and to compare results to previously published data.

**Methods:** Time-to-event analysis for mean treatment duration (estimated as the Restricted Mean Survival Time), b/tsDMARD failure, and b/tsDMARDs switching was performed for 230 patients (*n* = 147 RA, 46 PsA, 37 AS) who commenced their first b/tsDMARD between 2008 and 2018. Patients were managed in a dedicated “biologics” clinic in a tertiary hospital; the choice of b/tsDMARD was clinician driven based on medical factors and patient preferences. The effect of covariates on switching risk was analysed by a conditional risk-set Cox proportional-hazards model. Treatment retention data was compared to a historical analysis (2002–2008).

**Results:** The proportions remaining on treatment (retention) were similar, throughout follow-up, for the first, second and third b/tsDMARDs across all patients (*p* = 0.46). When compared to RA patients, the risk of b/tsDMARD failure was halved in PsA patients [Hazard Ratio (HR) = 0.50], but no different in AS patients (HR = 1.0). The respective restricted mean (95%CI) treatment durations, estimated at 5 years of follow-up, were 3.1 (2.9, 3.4), 4.1 (3.7, 4.6), and 3.3 (2.8, 3.9) years, for RA, PsA, and AS, respectively. Age, gender, disease duration, smoking status and the use of concomitant csDMARDS were not associated with the risk of bDMARD failure. The most common reasons for switching in the first and subsequent years were secondary (*n* = 62) and primary (*n* = 35) failure. Comparison with historical data indicated no substantive differences in switching of the first biologic for RA and PsA.

**Conclusion:** Similar retention rates of the second and third compared to the first b/tsDMARD in RA, PsA, and AS support a strategy of differential b/tsDMARDs use informed by patient presentation. Despite greater availability of b/tsDMARDs with differing mechanisms of action, retention rates of the first b/tsDMARD remain similar to previous years.

## Introduction

Biologic (b-) and targeted synthetic (ts-) disease-modifying anti-rheumatic drugs (DMARDs) represent a major therapeutic advance in rheumatology. However, response rates to b/tsDMARDs are variable (1); the evidence-base for directing switching from one agent to another is limited with most switches being empirical (2, 3); and, responses to b/tsDMARDs regardless of the mechanism of action are generally similar (4). Furthermore, the overall rates of sustained remission and improvement in function are relatively modest (5), owing to several potential factors including lack of established criteria to direct initial or subsequent choice of one b/tsDMARD over another with a different MoA, following failure or inadequate response.

The efficacy of a drug can be reliably determined by its retention rate or persistence (6, 7). Data from randomised control trials (RCTs) involving b/tsDMARDs is beset by limited trial duration, and switching, if allowed, is limited at the most to one another agent (8, 9). In addition, trials often have rigid entry criteria and relatively uniform patient populations with less co-morbidities, that cannot easily be generalised to real-life routine medical care (10, 11). In contrast, in the clinic, rather than “entry criteria” the b/tsDMARD choice is directed with due consideration of the diversity of patient presentation and patient-related contextual factors. Thus, although clinic data yield outcomes less impressive than those from RCTs (12), studies with real-life data are invaluable in providing significant insights in order to guide treatment individualisations and improve outcomes (13, 14).

Although international guidelines are available on the prescription of biologics in rheumatic disease, these do not generally recommend any single agent or class (15). The initial and subsequent choice of b/tsDMARD can be influenced by several variables which include route and frequency of administration, potential side-effects, co-morbidities and mandatory regulation requirements for concomitant conventional synthetic disease-modifying antirheumatic drug(s) (csDMARD).

Multiple studies, including real-life studies, have analysed cycling to another agent with the same MoA and also looked at switching to an alternative agent, in patients who have failed or have been intolerant to their current biologic agent (8, 16). The decades-long availability of TNFi with 5 agents in this class have led TNFi usually being the first biologic prescribed and to several studies assessing “cycling” within the same MoA. These studies revealed that using a second TNFi following the failure of the first TNFi can be an effective treatment approach (9, 17–19). However, larger and more studies appear to suggest that switching to an agent with a different MoA is more effective than cycling agents within the same class (6, 20–24). There is evidence to suggest greater retention of treatment (20, 22, 24, 25), lower chance of treatment failure (26) and a greater reduction in the disease activity (8, 24). Studies have also found that a change in MoA led to an improvement in physical function (27–29). Although these studies included real-life data, data on long-term retention rates and switching between the various b/tsDMARDs is limited. Furthermore, although there are some data suggesting greater retention of b/tsDMARDs in PsA vs. RA (30), few studies have assessed retention rates between RA, PsA, and AS; such data are expected to provide insights into comparative retention rates, effects of concomitant csDMARDs on retention rates, and in doing so, inform clinical practise and regulatory prescribing policy.

This study was conducted to assess retention rates of the first-, second-, and third-b/tsDMARDs in RA, PsA and AS in a tertiary hospital clinic setting. We also sought to compare b/tsDMARD retention rates between these diseases, assess the effect of concomitant DMARDs, explore trends of and reasons for switching, and compare with the historical data from this clinic.

## Methods

### The Australian Medicare/PBS System

The current prescription of b/tsDMARDs in Australia is mandated by the Australian Medicare system, which provides subsidised treatment at a uniform cost, regardless of the type of b/tsDMARD. A patient becomes “eligible” for a b/tsDMARD following failure of csDMARDs for RA and PsA, or for AS, failure of a trial of non-steroidal anti-inflammatory drugs. The choice of b/tsDMARD (apart from Rituximab, that mandates failure of a TNF inhibitor) is physician-driven and directed by patient demographics, comorbidities, and patient preferences. Specifically, to qualify for PBS subsidised b/tsDMARDs for RA and PsA, patient must have at least 20 active (both swollen and tender) or 4 active large joints. Patients who present with axial disease in the context of PsA are classified as AS. Patients with AS require to have radiographic sacroiliitis (grade 2 bilaterally or grade 3 unilaterally), and failure of at least 2 NSAIDs, to qualify for access to a b/tsDMARD. In Australia, bDMARDs were introduced for the treatment of rheumatic conditions around 2003, with Infliximab and Etanercept as the initial agents, followed by Adalimumab (2004), Abatacept and Rituximab (2007), Tocilizumab (2009), Golimumab and Certolizumab (2010), Ustekinumab and Secukinumab (2016). Tofacitinib, a synthetic small molecule Janus kinase (JAK) inhibitor, is a tsDMARD and was available in 2015.

### The “Biologics” Clinic

The Southern Adelaide Local Health Network (SALHN) in Adelaide, South Australia, comprises of three teaching hospitals offering rheumatological services, which include a dedicated, weekly, registrar-run and consultant-supervised, biologics clinic, established in 2002. Approximately 500 patients are enrolled in this clinic with the patient database being maintained by a dedicated “biologics” nurse. The majority of patients on b/tsDMARDs for RA, PsA, and AS, diagnosed within SALHN are referred to and managed in this clinic.

### Study Design

In this retrospective, longitudinal, observational study, we included all adult (>18 years) patients with RA, PsA, and AS, commencing first and subsequent b/tsDMARDs between July 1, 2008 and June 30, 2018. The diagnoses of RA, PsA and AS were made by their treating rheumatologist and at the time of enrolment, all patients were biologic naïve. We excluded patients if they did not receive or take b/tsDMARD treatment (despite enrolment), those with incomplete data or those who were subsequently lost to follow-up. Biologics for non-radiographic axial spondyloarthritis (nr-AxSpA) were not available on the PBS for most of the period covered by this study, and this group of patients were also excluded. This study was submitted to and approved by the Southern Adelaide Clinical Human Research Ethics Committee to proceed and to be published. However, because this study was designed for and intended to lead to iterative refinement in service provision, the Southern Adelaide Clinical Human Research Ethics Committee determined that it did not require a full formal ethical review.

### Data Capture

Data was collected retrospectively from the patient medical records. Data collected included demographics, duration of disease prior to commencing their first b/tsDMARD, csDMARD and prednisolone use, reasons for and dates of commencement and switching of b/tsDMARDs. Reasons for switching were classified as primary or secondary failure, adverse events, extra-musculoskeletal manifestations, comorbid conditions, infection or other.

Since there is no universally accepted consensus definition of primary and secondary failure, for the purposes of this study, primary failure was defined as not demonstrating efficacy with treatment within 3–6 months of treatment initiation and secondary failure as an initial response, subsequently lost on continued treatment. Persistence was defined as the time from initiation to discontinuation of biologic therapy.

Data was also compared to previous similar analysis undertaken in this centre prior to 2008 (31).

### Statistical Analysis

All statistical analysis was performed in Stata v16.1 (StataCorp LLC, TX, USA). The proportion of patients remaining on their prescribed b/tsDMARD therapy (retention) was analysed using time-to-event analysis methods for censored data to account for incomplete follow-up in patients without b/tsDMARD failure. Results were initially explored by Kaplan-Meier curves, but as patients experienced up to three b/tsDMARD failure events, further analysis was performed using conditional risk set time-to-event regression models for multiple failures (32).

Summary measures of the b/tsDMARD treatment retention curves over time included the %bDMARD retention and the mean treatment duration, both estimated at 5 years of follow-up. The mean treatment duration was estimated as the Restricted Mean Survival Time (RMST) (33), defined as the area under the treatment retention curve up to a fixed time t*, which estimated the failure-free treatment duration expectancy in the restricted follow-up period. The 25 and 50% centiles of the failure-time distribution were also determined. These estimated the treatment time by which 25 and 50% of patients had experienced b/tsDMARD failure, with the 50% centile being the overall median treatment duration. All summary measures were estimated from a spline-smoothed time-to-event regression model, which included covariates for both diagnosis and b/tsDMARD treatment episode, using the Stata user-defined (ado) programs “stpm2” (34) and “standsurv” (35). Overall summary estimates (for example, for each diagnosis) were obtained from the mean treatment retention curve derived from the three treatment episodes.

Point estimates of the mean treatment duration at 60 months (5 years) from previously published (31) historical data (for the first b/tsDMARD treatment only) were obtained by digitising the image of the Kaplan-Meier curves, using Digitizelt (v2.3.3 https://www.digitizeit.xyz/) software, and integration of the area under the curve using the trapezoidal rule.

Patient-level predictors for b/tsDMARD treatment failure were evaluated by a Cox regression model, with results reported as Hazard Ratios (HR). Reasons for b/tsDMARD treatment failure were compared using a competing risks analysis of the individual cause-specific cumulative incidence functions over time, estimated using the Stata ado program “stcompet” (36).

## Results

### Participants

The study included 230 patients: 147 RA (81% seropositivity), 46 PsA and 37 with AS (Table 1). The median age at diagnosis across all groups was 44 years, with a median disease duration of 6.4 years prior to commencement of their first biologic. As expected, there was a female predominance in RA patients (69%), and under-representation in AS (24%). The majority of the cohort (123, 53%) patients were either current or reformed smokers.

**Table 1:** Baseline demographics (at the time of the first b/tsDMARD) for rheumatoid arthritis (RA), psoriatic arthritis (PsA) and ankylosing spondylitis (AS) patients.

**Descriptor**	**RA**	**PsA**	**AS**	**All**
*N*	147	46	37	230
Female (%)	102 (69%)	26 (57%)	9 (24%)	137 (60%)
Diagnosis age (yrs): median (IQR)	48 (35, 60)	40 (31, 49)	37 (27, 45)	44 (29, 53)
Disease duration (yrs): median (IQR)	5.8 (3.6, 6.5)	5.2 (2.6, 7.6)	5.3 (1.9, 10.9)	6.4 (1.9, 10.9)
**Smoking**				
*Never*	59 (40%)	30 (65%)	18 (49%)	107 (47%)
*Reformed*	64 (44%)	13 (28%)	9 (24%)	86 (37%)
*Current*	24 (16%)	3 (7%)	10 (27%)	37 (16%)
Seropositive	119 (81%)			

### b/tsDMARD Treatment

The most common initial b/tsDMARD was a TNFi, prescribed in 109 (74%), 44 (96%), and 37 (100%) of RA, PSA, and AS patients, respectively. However, there was diversification in the class of newly prescribed b/tsDMARD over the second and third treatment episodes, with the proportion of patients prescribed another TNFi declining in all three diagnosis groups (Table 2). The most frequently prescribed TNFi were Etanercept (44%), Adalimumab (33%), and Golimumab (14%).

**Table 2:** Treatments, failures, retention rates and duration for each episode of b/tsDMARD use by diagnosis groups.

	**b/tsDMARD 1**	**b/tsDMARD 2**	**b/tsDMARD 3**
**Rheumatoid Arthritis**			
*N*	147	70	25
**b/tsDMARD:**			
*TNFi*	109 (74%)	33 (47%)	7 (28%)
*Tocilizumab*	22 (15%)	24 (34%)	6 (24%)
*Abatacept*	14 (10%)	7 (10%)	5 (20%)
*Tofacitinib*	1 (1%)	5 (7%)	3 (12%)
*Rituximab*	1 (1%)	1 (1%)	4 (16%)
Concomitant csDMARD (%)	95 (65%)	31 (44%)	11 (44%)
b/tsDMARD failures	72	28	7
Maximum follow-up (years)	9.9	7.7	7.4
Time at risk (person-years)	407.2	127.6	66.3
% Retention at 5 years (95% CI)	45 (37, 53)	47 (33, 59)	63 (39, 79)
Mean treatment duration at 5 years (95% CI)	3.1 (2.8, 3.4)	3.1 (2.6, 3.6)	3.7 (2.9, 4.4)
**Psoriatic arthritis**			
*N*	46	13	3
**b/tsDMARD:**			
*TNFi*	44 (96%)	10 (77%)	1 (33%)
*Secukinumab*	1 (2%)	1 (7%)	2 (67%)
*Ustekinumab*	1 (2%)	2 (1%)	0 (0%)
Concomitant csDMARD (%)	29 (50%)	7 (54%)	2 (100%)
Maximum follow-up (years)	10.0	9.2	6.0
b/tsDMARD failures	13	3	1
Time at risk (person-years)	175.7	33.0	6.2
% Retention at 5 years (95% CI)	69 (53, 80)	70 (50, 83)	80 (56, 92)
Mean treatment duration at 5 years (95% CI)	4.1 (3.6, 4.6)	4.1 (3.5, 4.7)	4.4 (3.9, 4.9)
**Ankylosing spondylitis**			
*N*	37	16	5
**b/tsDMARD:**			
*TNFi*	37 (100%)	15	3
*Secukinumab*	0	1	2
Concomitant csDMARD (%)	5 (14%)	1 (6%)	0 (0%)
b/tsDMARD failures	18	6	1
Maximum follow-up (years)	9.9	8.8	4.0
Time at risk (person-years)	124.9	45.3	5.6
% Retention at 5 years (95% CI)	52 (38, 64)	53 (36, 68)	68 (42, 84)
Mean treatment duration at 5 years (95% CI)	3.3 (2.7, 3.9)	3.3 (2.6, 3.9)	3.8 (2.8, 3.9)

**Figure 1:**
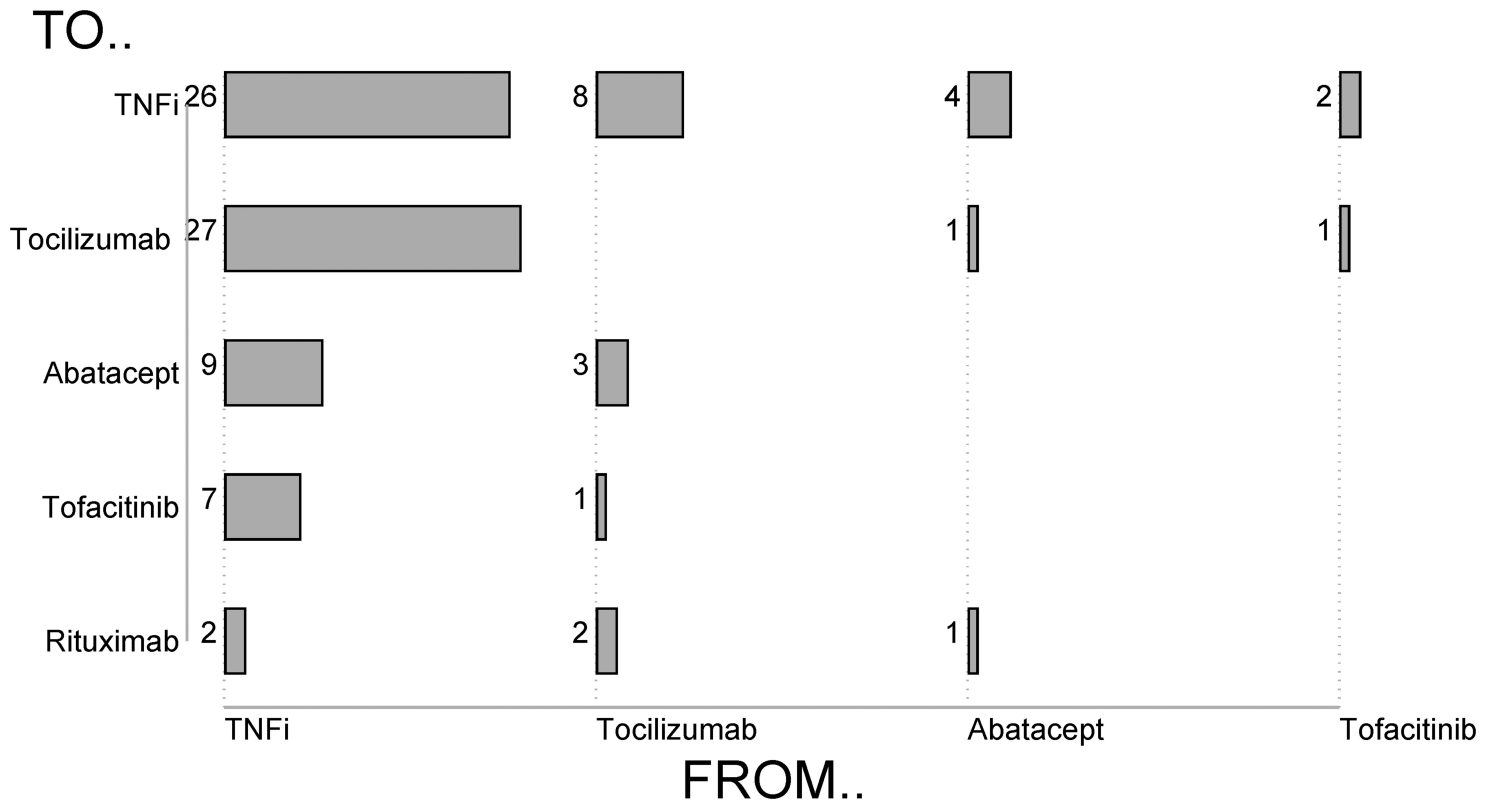
Types of b/tsDMARD switching in Rheumatoid Arthritis patients.

Tabulation of all b/tsDMARD switching events in RA (Figure 1) demonstrated that 41/71 (64%) of patients who failed a TNFi were switched to a non-TNFi b/tsDMARD predominantly tocilizumab. Conversely, 14/23 (61%) of RA patients who failed a non-TNFi b/tsDMARD were switched to a TNFi.

As expected, the majority of b/tsDMARD switching in both PsA and AS patients was within TNFi. In PsA, 3/16 patients who switched treatment switched from TNFi to secukinumab, and a further two switched to ustekinumab. In AS, 3/21 patients who switched treatment switched from TNFi to secukinumab.

### b/tsDMARD Retention/Failure

**Figure 2:**
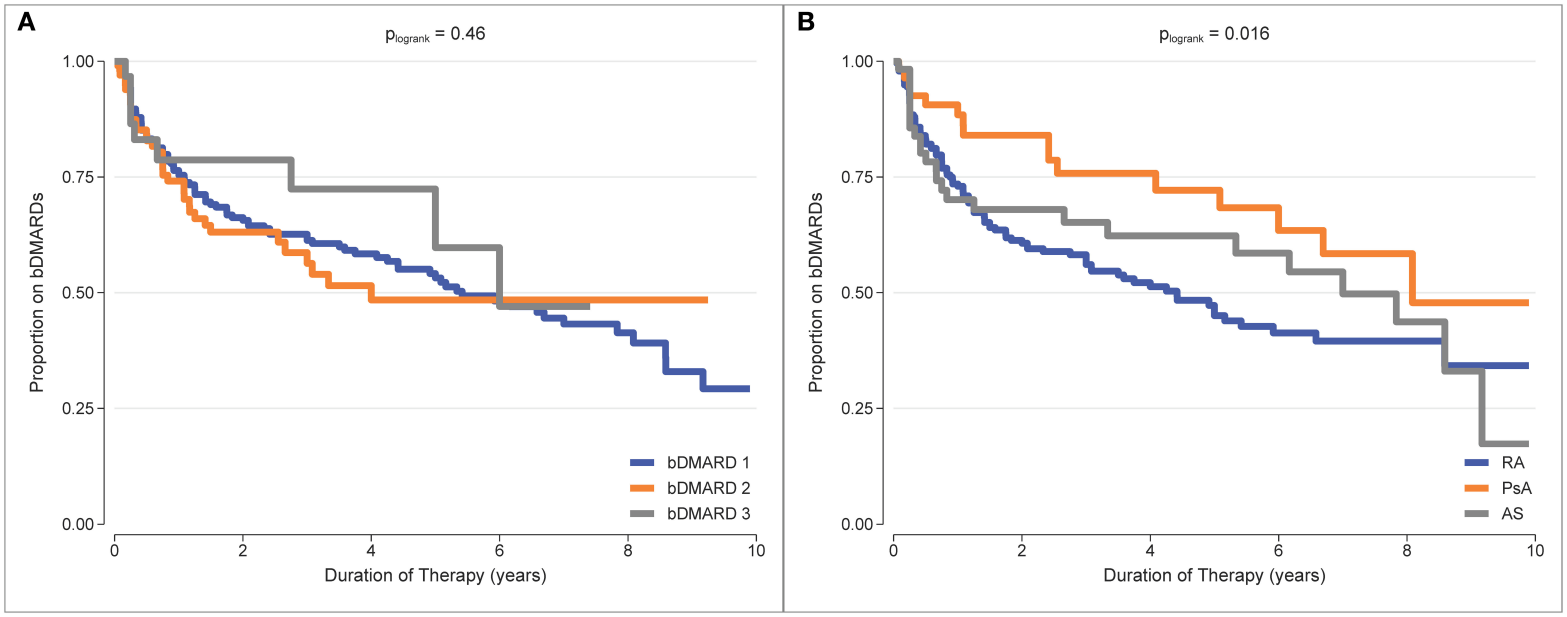
Kaplan-Meier curves for b/tsDMARD treatment retention. **(A)** b/tsDMARD treatment episodes (adjusted for diagnosis group). **(B)** Diagnosis group (adjusted for treatment episodes).

b/tsDMARD retention was estimated as the proportion remaining on treatment from Kaplan-Meier curves. Treatment retention and the restricted mean treatment duration at 5 years of follow-up for each treatment and each diagnosis are reported in Table 2. Examination of the Kaplan-Meier curves indicated that there was no difference in treatment retention between different treatment episodes (*p*_logrank_ test = 0.46, Figure 2A), indicating that initial treatment failure did not select for individuals who were subsequently inherently more likely to fail treatment. However, there was a difference in treatment retention by diagnosis (*p*_logrank_ test = 0.016, Figure 2B), with the best b/tsDMARD treatment retention observed in PsA patients.

A summary of b/tsDMARD retention for each diagnosis is reported in Table 3. At 5 years of follow-up, the overall treatment retention was 47% for RA, 70% for PsA and 53% for AS, and the overall median treatment duration before failure was 4.0, 8.2, and 6.2 years, respectively. Notably, 25% of b/tsDMARD failures in both RA and PsA patients occurred within the first year of follow-up.

**Table 3:** Summary measures from mean b/tsDMARD retention curves for rheumatoid arthritis (RA), psoriatic arthritis (PsA) and ankylosing spondylitis (AS) patients.

**Summary measure**	**RA**	**PsA**	**AS**	**ALL**
**At 5 years follow-up:**				
Mean b/tsDMARD duration (yrs)	3.1 (2.9, 3.4)	4.1 (3.7, 4.6)	3.3 (2.8, 3.9)	3.3 (3.1, 3.6)
% Retention	47 (41, 55)	70 (58, 85)	53 (42, 67)	52 (47, 58)
**Failure-time centiles (years):**				
25% Failures	0.8 (0.6, 1.2)	4.0 (1.9, 8.6)	0.9 (0.1, 6.5)	1.0 (0.6, 1.6)
50% Failures (median duration)	4.0 (2.6, 6.2)	8.2 (5.4, 12.4)	6.2 (3.7, 10.5)	5.5 (4.1, 7.5)

*Failure-time centiles define the treatment duration time by which the specified % of failures have occurred. Numbers in brackets represent 95% confidence intervals*.

Patient-level determinants of b/tsDMARD failure were explored by multivariable cox regression with results reported as hazard ratios (HR), (Table 4). When compared to RA patients, the risk of b/tsDMARD failure was halved in PsA patients (HR = 0.50), but no different in AS patients (HR = 1.0). Contrary to expectations, none of the additional covariates (age, gender, disease duration, smoking status and the use of concomitant csDMARDS) were associated with the risk of b/tsDMARD failure. Further, in an analysis restricted to RA patients, there was no relationship between seropositivity and b/tsDMARD failure (HR 0.89, 95% CI 0.53, 1.50, *p* = 0.66).

**Table 4:** Multivariable conditional risk set cox regression model (for multiple events) for predictors of b/tsDMARD failure.

**Covariate**	**HR (95% CI)**	***p*-value**
**Diagnosis group (base = rheumatoid arthritis)**		
Psoriatic arthritis	0.50 (0.27, 0.90)	0.022
Ankylosing spondylitis	1.00 (0.61, 1.64)	0.99
**Gender (base = male)**		
Female	1.14 (0.76, 1.69)	0.53
**Concomitant csDMARD (base = no)**		
Yes	1.19 (0.82, 1.71)	0.36
**Current smoker (base = no)**		
Yes	1.10 (0.70, 1.72)	0.69
Age (years)	1.00 (0.99, 1.01)	0.71
Disease duration (years)	1.01 (0.99, 1.02)	0.56

**Figure 3:**
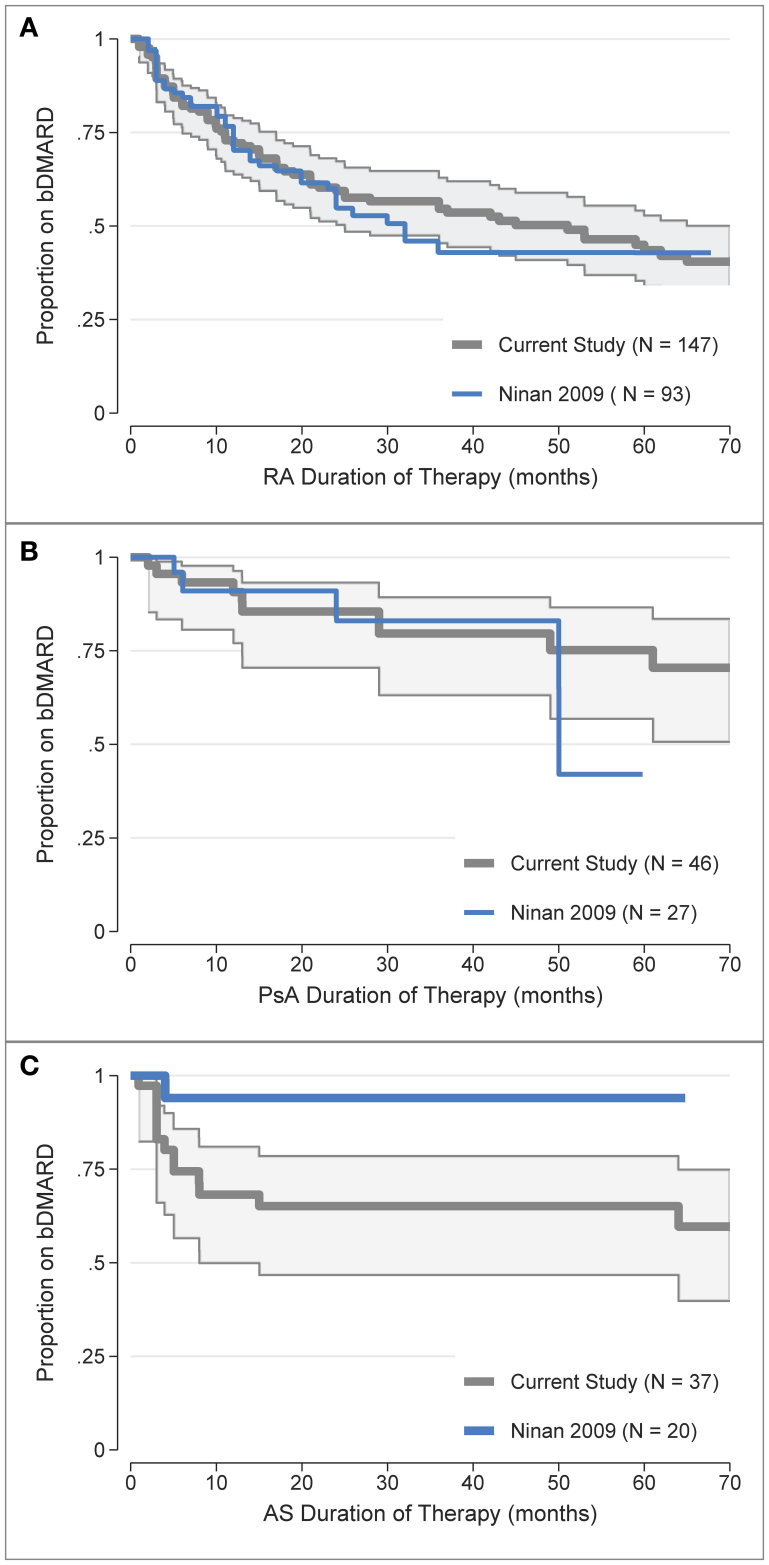
Comparison of the first b/tsDMARD treatment retention rates in the current study (post-July 2008) with earlier (pre-July 2008) data from Ninan et al. (31) for: **(A)** Rheumatoid Arthritis (RA), **(B)** Psoriatic Arthritis (PsA), **(C)** Ankylosing Spondylitis (AS). The digitised data from the Kaplan-Meier curves in Ninan et al. (31). Figure 1 was overlaid against the Kaplan-Meier treatment retention curves for the first b/tsDMARD in the current study, with shaded areas representing 95% confidence intervals.

*Results are expressed as hazard ratios (HR) with 95% confidence intervals*.

b/tsDMARD retention rates were also compared to historical, non-overlapping data from the same clinic (31), for which appropriate comparison data was only available for the first b/tsDMARD treatment. To enable comparison, the digitised data from the Kaplan-Meier curves reported in Figure 1 (31) for the first b/tsDMARD were overlaid against the Kaplan-Meier curves for the first b/tsDMARD in the current study (Figure 3). b/tsDMARD retention was comparable between the current and earlier study for both RA (Figure 3A) and PsA (Figure 3B). For RA, the point estimate of the restricted mean treatment duration at 60 months (5 years) in the historical study was 34.6 months (or 2.9 years), very similar to the current estimate of 3.1 at 5 years of follow-up reported in Table 2. Similarly, for PsA, the point estimate of the mean treatment duration at 60 months for the historical data was 48.0 months (4.0 years), again comparable to the current estimate of 4.1 years reported in Table 2. However, treatment retention for AS appeared somewhat worse in the current study, with the retention curve for the historical data, which had only one b/tsDMARD failure, clearly outside the confidence intervals for the current data (Figure 3C).

### Reasons for b/tsDMARD Failure/Switching

**Figure 4:**
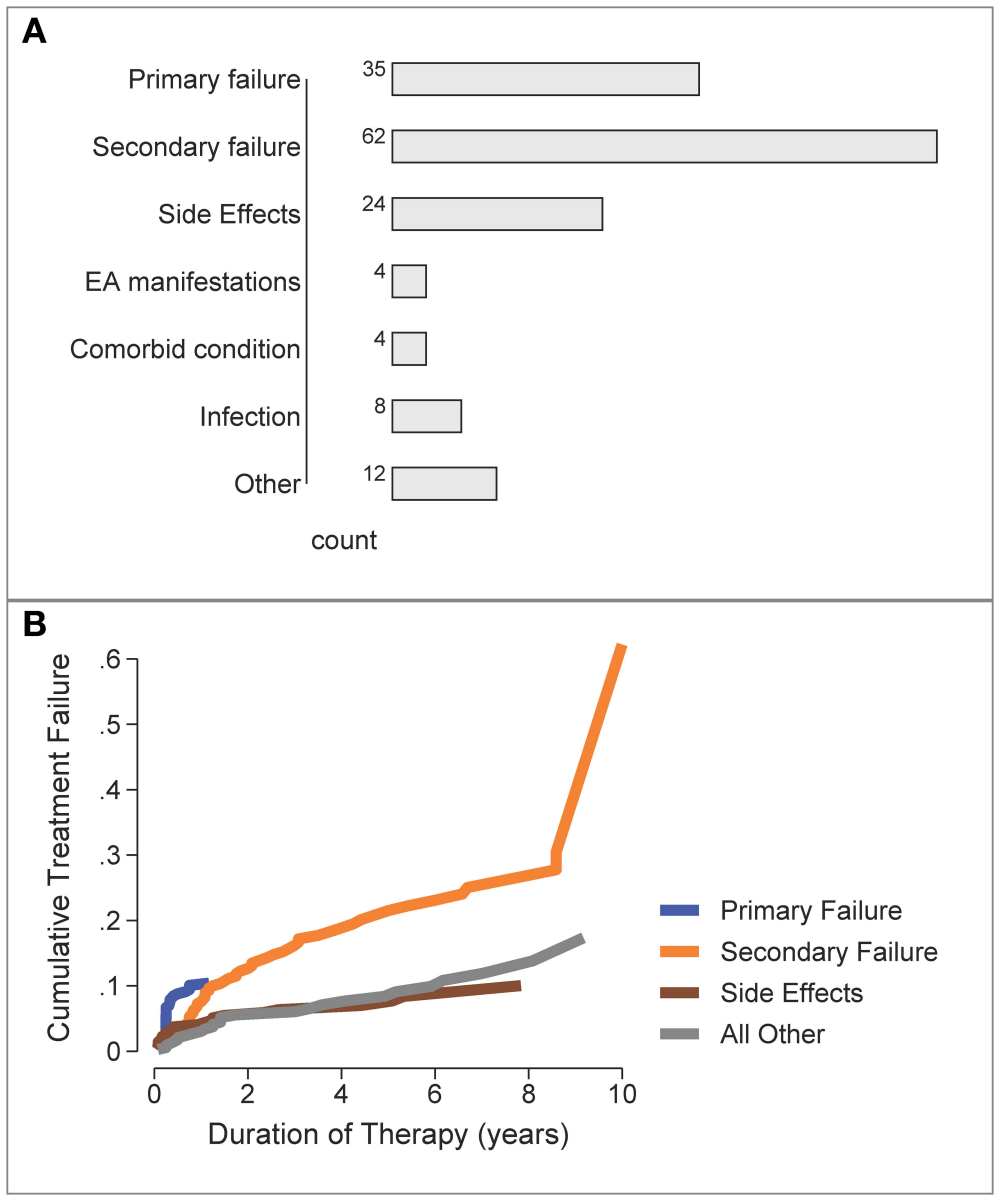
Reasons for b/tsDMARD failure over all patients and all treatment failures. **(A)** Frequency of specific failure types. EA, extra-articular/extra-musculoskeletal. **(B)** Cumulative Incidence of b/tsDMARD failure types over time (competing risks analysis).

The most frequent reasons for b/tsDMARD failure/switching were secondary failure (*n* = 62), primary failure (*n* = 35) and side effects (*n* = 24), Figure 4A. “Other” reasons for switching (*n* = 12) included pregnancy, malignancy and non-compliance.

The time-course for each type of treatment failure was examined by the cumulative incidence curve for each reason, estimated by a competing risks analysis (Figure 4B). Primary failure was the most likely reason for switching b/tsDMARDs during the first 12 months, with other reasons for b/tsDMARD failure/switching accruing over the longer term, with secondary failure accruing at the fastest rate.

## Discussion

In the absence of strict evidence-based established guidelines, biologic prescribing patterns vary across the world, directed by physician and patient preferences, and by b/tsDMARD availability and regulatory policy given the significant cost of b/tsDMARDs to funding bodies. Previous studies have analysed physician biologic prescribing patterns to better understand contemporaneous clinical practise (37–39). Erkan et al. (37) found that doctor preference and experience, and medication costs were important factors that influence treatment decisions. Other studies (40, 41) reflect the importance of patient preferences in b/tsDMARD choice, with regard to route of administration, frequency of dosing, tablet or injection formulation and previous experiences and drug-related adverse events. This was also taken in account in this study but medication costs however were not a factor given that these medications are not funded by the patient, but subsidised by the federal government PBS. Comparison between previous studies and ours is made difficult because of heterogeneity in b/tsDMARD availability, patient populations and contextual factors and regulatory policy; in addition, definitions of discontinuation and duration of follow-up are variable, with most studies limiting b/tsDMARD retention data to 1 year.

Our reported mean retention rates over 5 years of 47% for RA, 70% for PsA and 53% for AS and percentage retention for the first biologic (RA 45%, PsA 69%, and AS 52%) appear to be generally similar to those reported previously, with 12-month retention rates reported as 62.2–68.9% (42), 42–56% (43, 44), and 48–51% (45), and 5-year retention rate of 31–49% in a single study (46).

Data on the long-term retention of b/tsDMARDs in PsA and AS, in comparison to those in RA, are limited. In a study by Lyu et al. (47), bDMARD persistence ≥ 12 months was 57.9% for PsA, and 51.9 and 48.1% for RA and AS, respectively, generally reflecting our data, in contrast to a much lower proportion (36.1%) at 48 months, reported from the Corrona registry, of patients with PsA (48).

In a recent study by Murray et al. (30), b/tsDMARD retention rates for PsA were 58.9% at 1 year, and 52.3% at 12 years, with better retention rates with PsA compared to RA (49.6% at 1 year, 38.2% at 12 years). A significantly better retention rate was seen with PsA vs. RA in our current study also. Explanations for the higher retention rates in PsA vs. RA may be the earlier achievement of remission and possibly milder disease in PsA, as found in a study by Saber et al. (49) which showed that 58% of patients with PsA achieved remission with a TNFi compared to 44% of RA patients.

Retention rates of second and third b/tsDMARDs are more difficult to compare with our current study, as there are few studies that have analysed similar data. Two available studies on RA have reported results similar to ours with second b/tsDMARD (TNFi) retention rates of 46–56% (50) and 56.8% (51). In the latter study, the TNFi (vs. non-TNFi) group had a lower rate (53.5 vs. 66.7%) and retention was similar for different episodes of use. Previous studies have reported an inversely proportional relationship between drug survival and b/tsDMARD failure (6, 52–55) and we thus expected lower retention rates with subsequent episodes of b/tsDMARD use. This is also because tighter disease control is now the target of treatment, with earlier switching, especially in the setting of a greater number of agents now available (56).

One reason for the better than expected retention rates in our study with subsequent b/tsDMARDs may be our practise of switching to a b/tsDMARD with a different MoA, rather than to an alternative agent in the same class; this strategy is supported by previous studies (6, 20–25). With regard to TNFis specifically, several previous studies have found non-responders to TNFi therapy respond better to a b/tsDMARD with a differing mechanism of action as their next choice biologic (8, 23, 26).

Previous studies reflect our results of TNFi being the most commonly prescribed first line bDMARD (57). This is not surprising, as TNFi were the first class of b/tsDMARDs to be licenced for clinical practise, with much greater physician familiarity with efficacy, safety and short- and long-term potential adverse events. In our study, appropriately, in those that required to be switched to their second and third b/tsDMARDs for RA, there was an increase in switching to a different class of agents—usually tocilizumab and abatacept.

Unexpectedly, we did not find an association between b/tsDMARD failure and concomitant csDMARD use in RA, in contrast to previous studies that have demonstrated superior b/tsDMARD retention rates in RA, in particular with TNFis, with concomitant csDMARDs (58–60). In contrast to the aforementioned studies, Bechman et al. (61) found that TNFi monotherapy had equivalent retention to TNFi-csDMARD combination therapy in patients >75 years, perhaps as a result of immunosenescence. In our study however, only 17.7% were over 75 years.

An increased use of tocilizumab, often as monotherapy without csDMARDs, especially during class switching, may also have contributed to the lack of an association between b/tsDMARD failure and concomitant csDMARD use in our study. Previous studies have demonstrated the equivalent efficacy (62) and retention rates (63, 64) of Tocilizumab monotherapy as the first-line or subsequent bDMARD. We do acknowledge however that had there been increased use of Tofacitinib, there may have been a change in the results given that Tofacitinib has a synergistic effect when combined with methotrexate (65, 66). We aim to assess this in the next iteration of this study by which time we expect to have higher number of patients on JAK inhibitors.

We also did not find any significant association with b/tsDMARD failure with female gender, smoking, age, and disease duration, or seropositivity. Courvoisier et al. (67) recently published a pooled analysis of observational data, of those on treatment with Rituximab, Abatacept, Tocilizumab or TNFi, and demonstrated greater effectiveness of non-TNFi bDMARDs, especially Rituximab and Abatacept (vs. TNF) in seropositive patients with RA. This association with Abatacept response and seropositive RA has been reported in other studies (68, 69). Smoking is associated with a higher disease activity and lower retention of bDMARD, which is in line with previous studies (55, 70). Our study did not show any association between bDMARD retention and smoking and this could be attributed to a significant proportion of patients in this study having never smoked (47%) and only a small percentage (16%) as current smokers.

Existing literature confirms our findings of inefficacy (primary or secondary) as the most common reason for discontinuing or switching bDMARD therapy, followed by adverse events to the agent (7, 29, 71–73).

We did not find significant differences in the first bDMARD retention over time, as compared to previously published data from our clinic (31). This was contrary to our expectation of earlier and more frequent switching in recent years in order to achieve tighter disease control and with the availability of multiple new b/tsDMARDs with differing MoA. Our results contrast to those reported by Du Pan et al. (52), who found an inversely proportional relationship with initiating biologic treatment in a later year and drug survival, which they attributed to greater availability of the number of b/tsDMARDs. Our results are probably explained by the ongoing practise of TNFi being the most common initial bDMARD, similar to the previous study from this clinic, although at that time, few other classes of b/tsDMARDs existed.

The strengths of this study lies in it being a real-world study representing contemporaneous clinical practise, rather than a RCT. Additionally, it is one of very few studies comparing data from three inflammatory arthritides, over a 10-year period, with a follow-up period of minimum 5 years, and with an added advantage of comparison with previous data from the same centre, enabling observations of changes in trends over time.

The limitations of this study are its observational nature with relatively limited patient numbers and use of retrospectively collected data from patient medical records, that relied upon accurate documentation, which was not always present and led to those patients being excluded from the analysis. Although AS and PsA have a similar prevalence in Australia as compared to the rest of the world, the specialised clinic that this data is drawn from receives patient referrals from the Southern region of Adelaide, which may be a reason as to why sample size was limited; in addition, the Australian subsidised PBS criteria for access to biologics are relatively strict and inflexible. The latter reason means that some patient who would clinically benefit from addition of a b/tsDMARD are unable to access these drugs.

In the absence of randomisation, patients may have been guided to a specific drug, producing selection bias. Additionally, in this long-term drug survival analyses, the number of patients at risk progressively decreased by time, being lower at the end of the evaluated follow-up period and this trend may partially influence the results. Some b/tsDMARDs were not available until later in the period and therefore their penetration in this study may have been limited.

## Conclusion

Similar retention rates of the second and third compared to the first b/tsDMARD support a strategy of differential b/tsDMARD use informed by patient presentation. Despite greater availability of b/tsDMARDs with differing mechanisms of action, retention rates of the first b/tsDMARD remain similar to previous years.

## Data Availability Statement

The original contributions presented in the study are included in the article/supplementary material, further inquiries can be directed to the corresponding author.

## Ethics Statement

The studies involving human participants were reviewed and approved by Southern Adelaide Clinical Human Research Ethics Committee. Written informed consent for participation was not required for this study in accordance with the national legislation and the institutional requirements.

## Author Contributions

MW contributed to conception and design of the study. LB organised and maintained the database. VB and RH collected and entered patient data. SL performed the statistical analysis and wrote the results section. VB wrote the first draught of the manuscript. MW, SL, and VB wrote sections of the manuscript. All authors contributed to manuscript revision, read, and approved the submitted version.

## Conflict of Interest

The authors declare that the research was conducted in the absence of any commercial or financial relationships that could be construed as a potential conflict of interest.

## Publisher's Note

All claims expressed in this article are solely those of the authors and do not necessarily represent those of their affiliated organizations, or those of the publisher, the editors and the reviewers. Any product that may be evaluated in this article, or claim that may be made by its manufacturer, is not guaranteed or endorsed by the publisher.
